# Effects of corn steep liquor on β-poly(l-malic acid) production in *Aureobasidium melanogenum*

**DOI:** 10.1186/s13568-020-01147-8

**Published:** 2020-12-01

**Authors:** Genan Wang, Bingyi Shi, Pan Zhang, Tingbin Zhao, Haisong Yin, Changsheng Qiao

**Affiliations:** 1 Key Laboratory of Industrial Fermentation Microbiology (Tianjin University of Science and Technology), Ministry of Education, Tianjin, 300457 People’s Republic of China; 2 Tianjin Engineering Research Center of Microbial Metabolism and Fermentation Process Control, College of Biotechnology, Tianjin University of Science and Technology, Tianjin, 300457 People’s Republic of China; 3 School of Bioengineering, Tianjin Modern Vocational Technology College, Tianjin, 300350 People’s Republic of China; 4 Tianjin Huizhi Biotrans Bioengineering Co., Ltd., Tianjin, 300457 People’s Republic of China

**Keywords:** β-poly(l-malic acid) (PMLA), Corn steep liquor, *Aureobasidium melanogenum*, Metabolomics

## Abstract

β-poly(l-malic acid) (PMLA) is a water-soluble biopolymer used in medicine, food, and other industries. However, the low level of PMLA biosynthesis in microorganisms limits its further application in the biotechnological industry. In this study, corn steep liquor (CSL), which processes high nutritional value and low-cost characteristics, was selected as a growth factor to increase the PMLA production in strain, *Aureobasidium melanogenum*, and its metabolomics change under the CSL addition was investigated. The results indicated that, with 3 g/L CSL, PMLA production, cell growth, and yield (Y_*p/x*_) were increased by 32.76%, 41.82%, and 47.43%, respectively. The intracellular metabolites of *A. melanogenum*, such as amino acids, organic acids, and key intermediates in the TCA cycle, increased after the addition of CSL, and the enrichment analysis showed that tyrosine may play a major role in the PMLA biosynthesis. The results presented in this study demonstrated that the addition of CSL would be an efficient approach to improve PMLA production.

## Key points

- CSL can benefit the β-poly(l-malic acid) production.
- CSL influences the metabolism of the amino acids.
- CSL could be used as an economic nitrogen source for PMLA production.

## Introduction

Polymalic acid (PMLA) is a polyester of l-malic acid with a wide range of applications in the medical, food, and environmental industries due to its excellent biochemical properties, including biocompatibility, biodegradability, and chemical modifiability (Zeng et al. 2019). Several chemical synthesis routes are available in the production of PMLA (Kajiyama et al. 2004; Portilla-Arias et al. 2008; Vert 1998), but these methods are costly, polluted, and difficult to scale up for commercial applications. Microorganisms, such as *Aureobasidium melanogenum*, can also produce PMLA from sugar during fermentation at high production rates, optical purity, and high molecular weights suitable in many applications (Zou et al. 2019).

Regardless of the microorganism used in PMLA production, l-malic acid is the only precursor in PMLA biosynthesis (Zeng et al. 2019). The three major metabolic pathways in PMLA biosynthesis are the tricarboxylic acid cycle (TCA), reductive TCA (rTCA), and glyoxylate pathway (Chi et al. 2016). In recent years, several factors were tested to increase PMLA production, including the screening of mutant strains, optimizing fermentation conditions, selecting suitable carbon sources, and adding growth factors (Cao et al. 2019a, b). Moreover, the researcher speculated that the metabolic pathway of PMLA may vary in different strains. The research found that PMLA production is considerably associated with the glyoxylate pathway in *Physarum polycephalum* with the addition of intermediates and inhibitors (Lee et al. 1999), and it can also be catalyzed by nonbiotin-dependent carboxylation, which is related to the rTCA pathway in *A. pullulans *(Cao et al. 2014). Due to the varied biosynthesis pathways for PMLA production, it is challenging to construct genetically engineered strains. As a result, selecting a suitable growth factor becomes an easy way to improve PMLA production.

Corn steep liquor (CSL) is a by-product of the corn wet-milling industry that contains nutrients essential for microbial growth. It has been proposed as a potentially effective substrate for many target metabolites produced by microorganisms (Amado et al. 2017). Researchers found that CSL increases the production of citric acid and calcium malate in *Yarrowia lipolytica* (Cavallo et al. 2020) and *Penicillium viticola* (Khan et al. 2014), respectively. However, in recent years research of PMLA biosynthesis, little attention has been paid to investigate the mechanism from a metabolomics aspect.

Gas chromatography–mass spectrometry (GC–MS) is a widely used analytical technique with high separation efficiency and sensitivity detection in resolving complex biological mixtures (He et al. 2018). Moreover, the intracellular metabolites produced by microorganisms influenced by different growth factors could be detected via GC–MS. The researcher investigated the various effects of deregulating enzymes on the metabolites between engineered l-lysine-producing *Corynebacterium glutamicum* and the wild-type strain through intracellular metabolite profiles (da Luz et al. 2017). Some other researchers combined intracellular metabolites with proteomics to identify the difference between marine sediment (Beale et al. 2017) and trace pollution (Beale et al. 2018). This approach could easily detect the main difference between microorganisms under different conditions or growth factors.

In this study, we aimed to explore the effect of CSL on the enhancement of cell growth and PMLA production in *A. melanogenum*. Metabolomics technology was used to gain insight into the working mechanism and to analyze the change in the key intracellular metabolites of *A. melanogenum* after CSL addition. The results will help to determine an efficient approach to improve PMLA production.

## Materials and methods

### Microorganism and medium

*Aureobasium melanogenum* CGMCC18996 was isolated in our laboratory and then preserved in the China General Microbiological Culture Collection Center (Beijing, China No. CGMCC18996). The strain was stored in potato dextrose agar (PDA) slants at 4 °C and subcultured every 2 weeks. The seed medium contained 60 g/L sucrose, 3 g/L yeast extract, 2 g/L succinic acid, 1 g/L ammonium sulfate, 0.4 g/L K_2_CO_3_, 0.1 g/L KH_2_PO_4_, 0.1 g/L MgSO_4_, 0.05 g/L ZnSO_4_, and 0.1% CSL (V/V). The fermentation medium contained 180 g/L sucrose, 35 g/L peptone, 0.1 g/L KH_2_PO_4_, 2 g/L NaNO_3_, 0.3 g/L MgSO_4_, 0.5 g/L KCl, 0.05 g/L MnSO_4_, and 20 g/L CaCO_3_. Both seed and fermentation mediums were sterilized at 121 °C for 20 min before use.

The amino acids and vitamins in CSL were determined via the methods of Culea et al. (2015) and Klejdus et al. (2004).

### Fermentation conditions

The primary seed culture of *Aureobasidium. melanogenum* CGMCC18996 was prepared by inoculating cells grown on solid medium into 500 mL Erlenmeyer flasks containing 100 mL seed culture medium and then cultured at 25 °C for approximately 40 h in a rotary shaker (IS-RDS3, Crystal Technology and Industries, Inc., USA). CSL at 1, 3, 5, 7, and 9 g/L was placed into 500 mL Erlenmeyer flasks containing 100 mL fermentation medium with primary seed culture (10%, v/v), and fermentation cultivation was conducted at 25 °C for 144 h in a rotary shaker at 200 rpm. Fed-batch fermentation kinetics was investigated in a 5 L stirred tank fermenter (GRJB-5D, Zhenjiang Gree Co., Ltd., China) containing 3 L fermentation medium inoculated with 300 mL seed culture, and the fermentation medium was operated at 25 °C for 156 h with an agitation speed and aeration rate of 500 rpm and 1.3 vvm, respectively.

### Assay of PMLA production

Fermentation broth (10 mL) was collected at different time points and centrifuged at 10,000×*g*. The resulting supernatant (5 mL) was mixed with 5 mL 2 M H_2_SO_4_ and then incubated at 110 °C for 11 h. After neutralization, the sample was analyzed with HPLC (L-2000, Hitachi Ltd., Japan) by using a Prevail C18 organic acid column at 25 °C eluted with 25 mM KH_2_PO_4_ at a rate of 1.0 mL/min. The PMLA concentration was determined by comparing the difference in l-malic acid concentrations before and after hydrolysis.

### Assay of fermentation parameters

Cell density was determined via the method of dry cell weight (DCW) in three steps. Prior to measurement, HCl (3 M) was added to 10 mL of fermentation broth to eliminate the excess concentration of CaCO_3_. The fermentation broth (10 mL) was centrifuged at 8000×*g* for 10 min, and the resulting precipitate was washed twice with phosphate buffer saline (PBS) buffer. After recentrifugation, the precipitates were dried overnight at 80 °C and then weighed.

The residual sugar was analyzed with the 3,5-dinitrosalicylic acid assay (Miller 1959).

### Specific growth rate, PMLA productivity, and PMLA yield (Y_p/x_) calculation

The specific growth rate was measured using the increased biomass versus interval time (Liu et al. 2005), and Y_*p/x*_ was measured by determining the ratio of increased PMLA to the increased cell biomass concentration over the interval time (Yin et al. 2019).

### Extraction of intracellular metabolites and metabolomics analysis

In the extraction of intracellular metabolites, three independent fermentation broth samples (50 mL) at the fermentation times of 72, 96, 120, and 144 h were collected from the 5 L stirred tank fermenter. The excess concentration of CaCO_3_ that was not consumed by *A. melanogenum* was removed by centrifuging the samples at 8000×*g* for 30 s. The resulting supernatant was centrifuged at 10,000×*g* for 5 min. The precipitate was collected, washed twice with normal saline at − 4 °C, and ground in liquid nitrogen for 25 min. The cell fragment (200 mg) of liquid nitrogen grind was collected and mixed with 1 mL of precooling methanol (60%). The mixture was then centrifuged at 8000×*g* for 5 min. After the derivatization process, it was subjected to refrigerated centrifugation at 8000×*g* for 5 min, and the resulting supernatant was prepared for GC–MS analysis after storage for 2 h at 25 °C.

### Data processing and analysis

GC–MS files (.MS) were converted to AIA (.CDF) format for XCMS online (Gowda et al. 2014) analysis. Peak detection and alignment were measured by default centwave method for GC Single Quadruple (Agilent 7980A/5975C, GC-MSD), and the METLIN database was used for Metabolites identification.

GC–MS pre-processing data were written as .csv files and imported to the MetaboAnalyst (Chong et al. 2019) for data normalization. The peak intensity represented the relative concentration, and the downstream analysis was performed by R studio using package BiocManager version 1.30.10.

## Results

### Effect of CSL addition on PMLA production of *A. melanogenum*

**Fig. 1:**
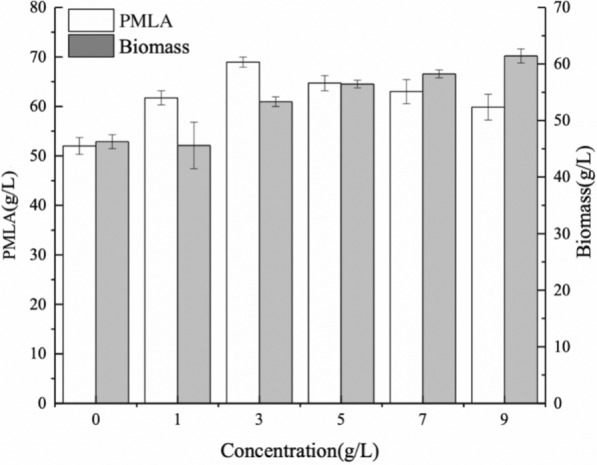
Different concentrations of CSL on PMLA production and biomass

**Fig. 2:**
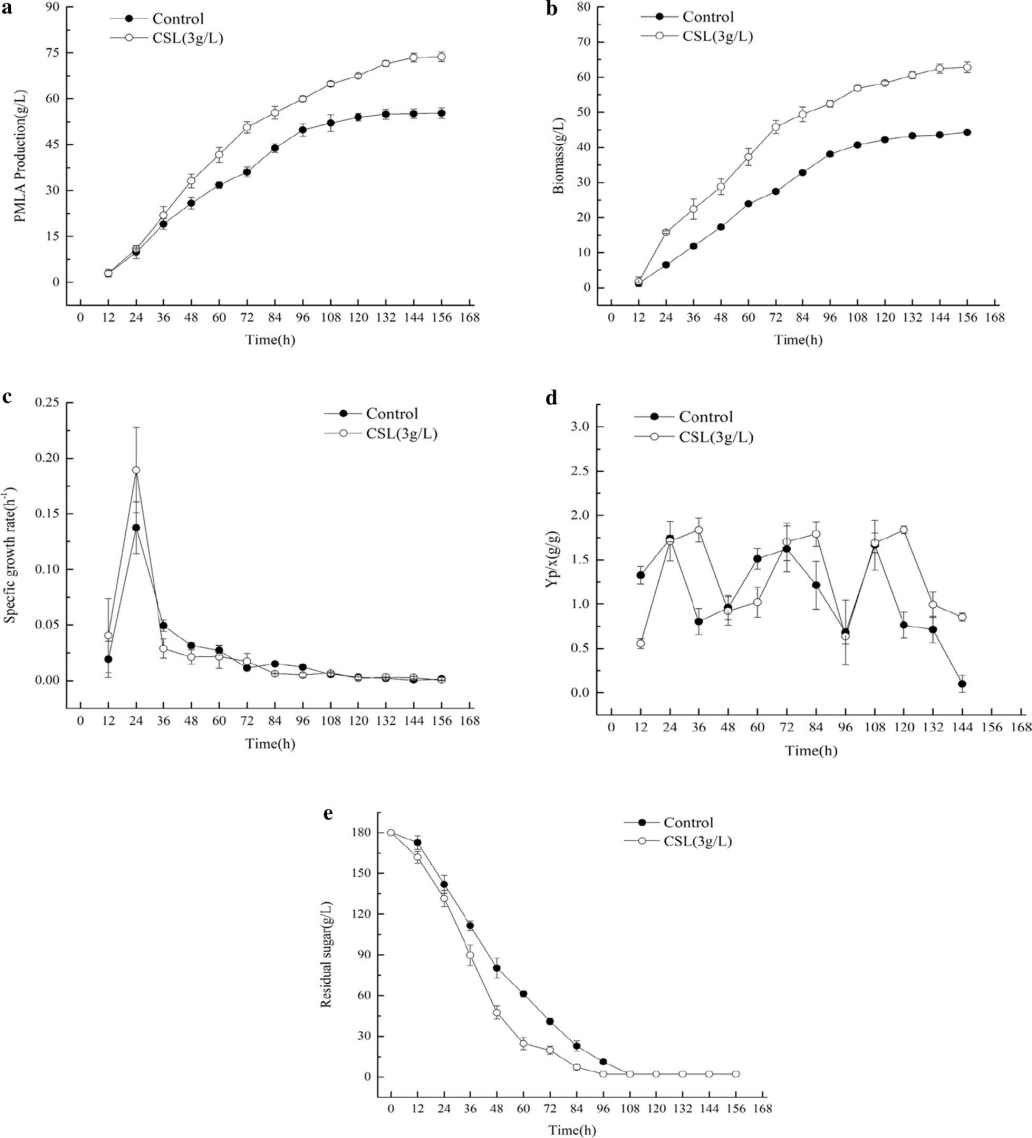
PMLA production versus **a** biomass, **b** specific productivity rate, **c** specific growth rate, **d** PMLA yield, and **e** residual sugar in the 5 L fermenter

In this study, the effects of different CSL concentrations on PMLA production of *A. melanogenum* that cultured in a rotary shaker were evaluated. The accumulation of biomass increased with the increasing CSL concentration, and the highest PMLA production, 69.8 g/L, was obtained with the addition of 3 g/L CSL, revealing a 36.8% increase compared to the control (Fig. 1). The effect of CSL on the PMLA production of *A. melanogenum* was further verified by culturing *A. melanogenum* in a 5 L fermenter (Fig. 2), and the results indicated that the addition of 3 g/L CSL facilitated the growth and PMLA production of *A. melanogenum.* After 156 h of fermentation, PMLA production reached 73.72 g/L, as shown in Fig. 2a, which was 32.76% higher than that of the control group. The biomass reached 62.83 g/L, as shown in Fig. 2b, which was 41.82% higher than that of the control group. Meanwhile, after 24 h of fermentation, the highest specific growth rate reached 0.19 h^−1^, as shown in Fig. 2c, which was 37.72% higher than that of the control group. However, a rapid decline in the specific growth rate was detected after 24 h when no distinct difference existed between the control and CSL groups. The PMLA yield (Y_*p/x*_) in CSL showed a significant increase in the late stage of fermentation, as shown in Fig. 2d. After 120 h, the highest Y_*p/x*_ reached 1.82 g/g, which was 47.43% higher than that of the control group. Moreover, the residual sugar (Fig. 2e) was consumed rapidly after the addition of 3 g/L CSL, and the end time was 12 h earlier than that of the control.

### Metabolomics analysis of *A. melanogenum* cultivated with the addition of 3 g/L CSL

**Fig. 3:**
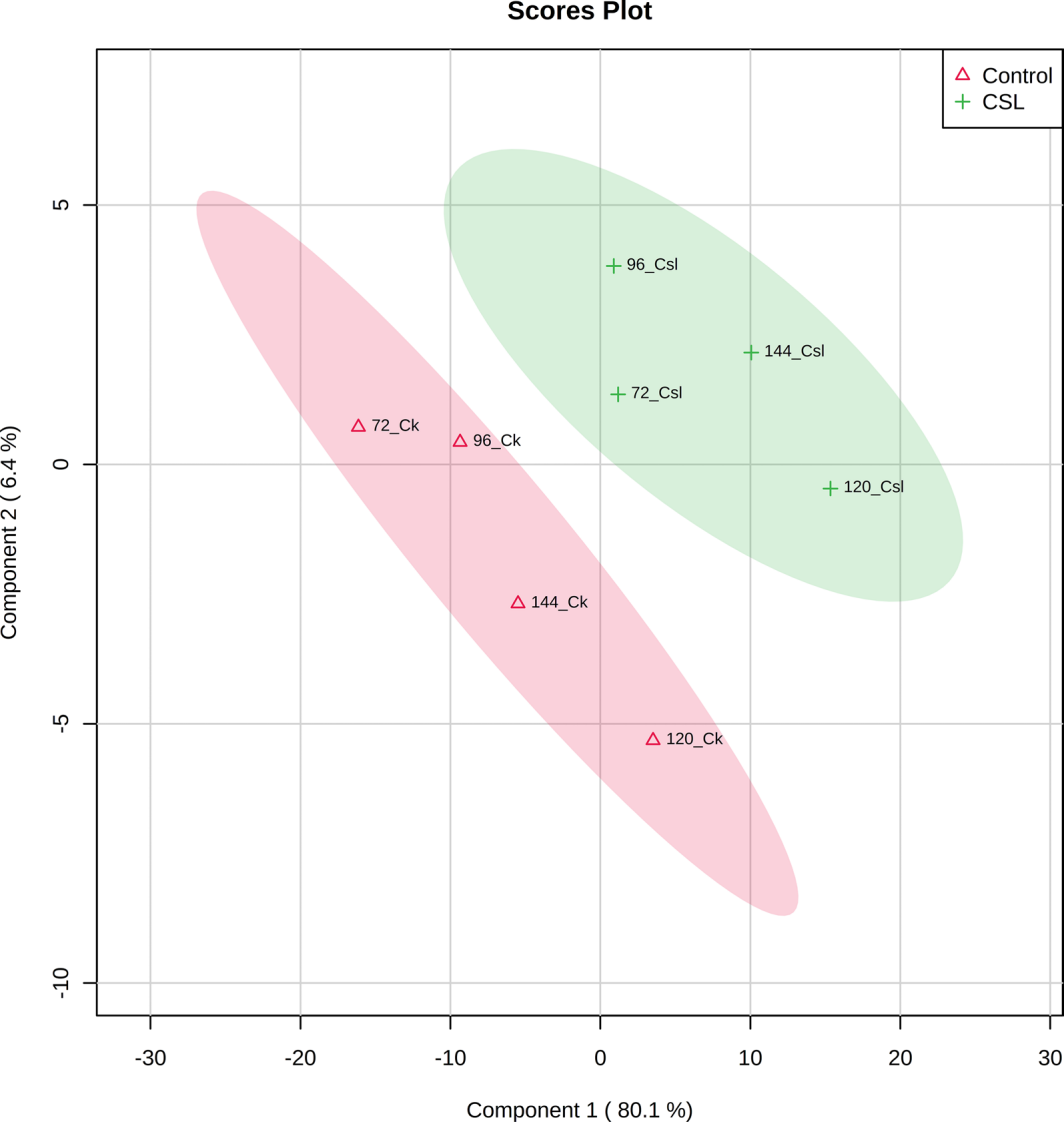
PLS-DA score plot of the control and CSL

**Fig. 4:**
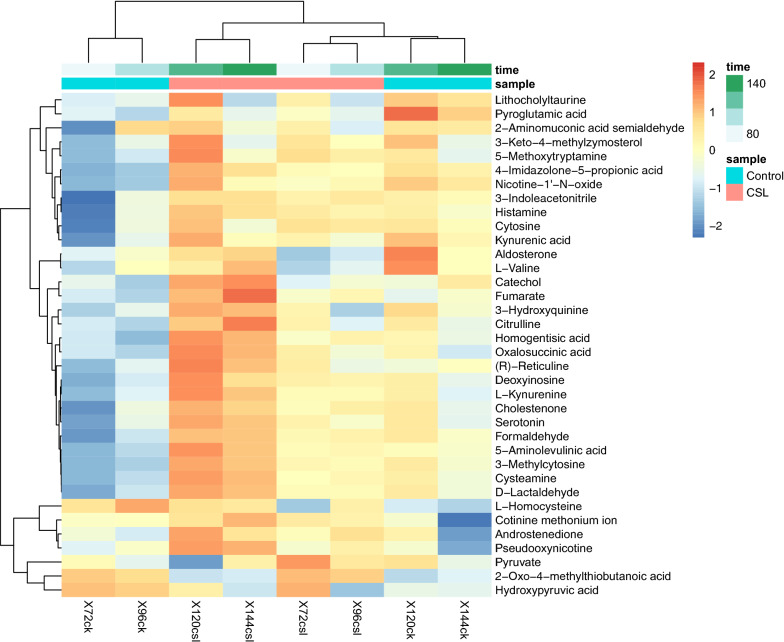
Heat map of the relative concentrations of metabolites in the control and CSL

The PLS-DA scores plot (Fig. 3) showed a clear variation in the metabolite profiles under both groups, and the metabolomics data revealed a total of 36 metabolites of *A. melanogenum* that were detected via GC–MS at 72, 96, 120, and 144 h time points (Fig. 4). Among them, most of the metabolite concentrations in the CSL group were increased compared to the control, especially at 120 h (Fig. 4). The enrichment analysis (Additional file 1: Figure S1) indicated that the concentrations of metabolite related to PMLA biosynthesis increased obviously. These metabolites were deoxyinosine, homogentisate, fumarate acid, and 5-aminolevulinic, which involved in the purine metabolism (P < 0.05), tyrosine metabolism (P < 0.05), TCA cycle (P < 0.05), and glycine and serine metabolism.

### Metabolomics analysis of PMLA metabolic pathway

**Fig. 5:**
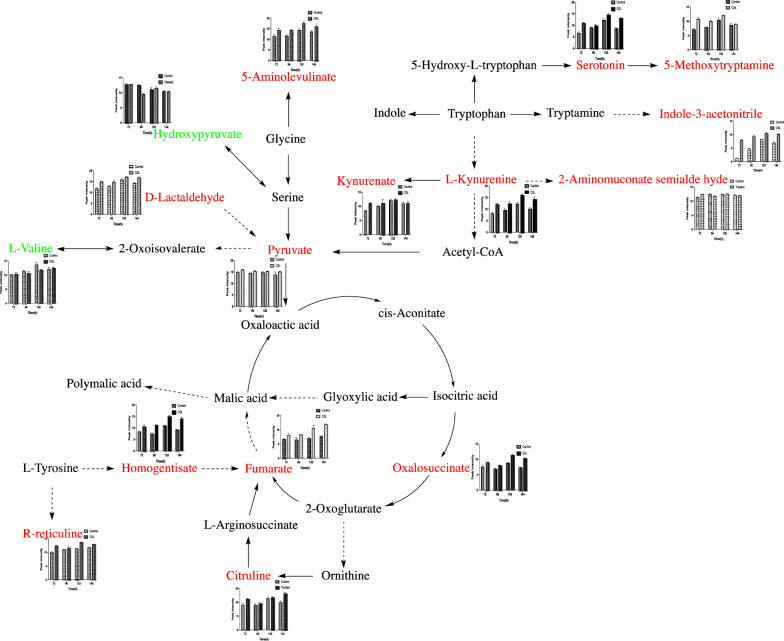
Metabolic pathway related to the PMLA biosynthesis based on the metabolomics data (color in red and green represent up-regulated and down-regulated metabolites)

The metabolic pathway related to PMLA biosynthesis and the relative concentration changes were depicted (Fig. 5). Compared to the control, nearly all of the metabolite concentrations increased and peaked at 120 h. The concentrations of six metabolites related to the tryptophan metabolism, which were serotonin, 5-methoxytryptamine, indole-3-acetonitrile, 2-aminomuconate semialde hyde, l-kynurenine, and kynurenate, were improved. The addition of CSL caused the accumulation of 5-aminolevulinate that involved in the glycine and serine metabolism. Moreover, the concentrations of homogentisate and R-reticuline, which related to the tyrosine metabolism, and d-lactaldehyde that would enter the TCA cycle were increased. For the TCA cycle, the concentrations of fumarate and oxalosuccinate, which are the precursors of malic acid, showed an increasing effect. And the results also indicated that the concentrations of l-valine and hydroxypruvate were decreased.

### Assay of the amino acids on PMLA production

**Fig. 6:**
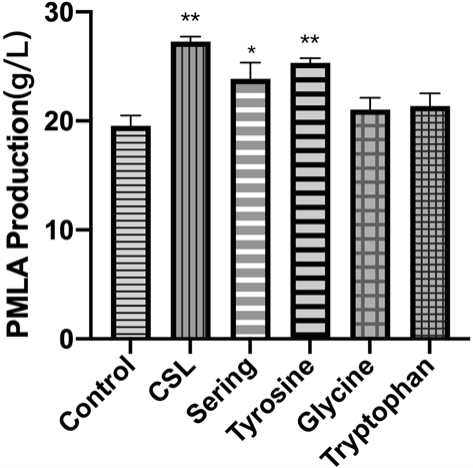
Different amino acids on PMLA production (^*^P < 0.05, ^**^P < 0.01 versus the control group by the LSD analysis)

The nutritional substances of CSL were tested. Seventeen kinds of amino acids and three types of vitamins were detected. Table 1 showed the ratio of each amino acid quality to the CSL quality. Among them, four amino acids (tyrosine, serine, glycine, and tryptophan) were selected based on the PMLA biosynthesis pathway (Fig. 5) and added to the fermentation broth (without peptone) separately for evaluating the influence on PMLA production. The result revealed that the PMLA production increased by 29.5% after adding tyrosine, 21.9% after adding serine, 9.3% after adding tryptophan, 7.6% after adding glycine, and 39.47% after adding CSL (Fig. 6). Moreover, significant changes were observed between the tyrosine group and the control (P < 0.01), and the CSL and the control (P < 0.01).

**Table 1:** Nutritional substances contained in the corn steep liquor

Name	Unit	Test result	Name	Unit	Test result
Amino acids					
Aspartic acid	%	2.22	Alanine	%	3.84
Threonine	%	1.20	Valine	%	1.98
Serine	%	1.28	Methionine	%	0.72
Glutamate	%	5.41	Isoleucine	%	1.21
Glycine	%	2.05	Leucine	%	3.40
Tyrosine	%	0.62	Phenylalanine	%	1.38
Lysine	%	1.16	Histidine	%	1.16
Arginine	%	1.51	Proline	%	3.20
Tryptophan	%	0.17			
Vitamins					
Vitamin B_2_	mg/kg	11.40	Choline	mg/100 g	344
Inositol	mg/100 g	226

## Discussion

Using the growth factor is an economical way to improve PMLA production (Cao et al. 2019b). In this study, the addition of 3 g/L CSL facilitates cell growth and PMLA production in *A. melanogenum*. The maximal PMLA production and biomass increased by 32.76% and 41.82%, respectively. Furthermore, the specific growth rate revealed a rapid increase during the early phase of fermentation, and Y_*p/x*_ in the CSL group was higher than that of the control in the late stage. Moreover, CSL contained various amino acids and vitamins (Table 1) that could function as an effective stimulatory agent of cell growth and PMLA biosynthesis. It was found that the rich nutrients of CSL became a source of nitrogen, essential minerals, and cofactors required for *Pichia pastoris* cell growth (Zheng et al. 2012). Moreover, the addition of CSL increased the biomass of *Trametes versicolor* in the initial 12 h of fermentation, and an accelerated growth rate was observed (Wang et al. 2014). The addition of 3 g/L CSL provided various amino acids and vitamins (Table 1), indicating that the CSL could be an effective stimulatory agent of cell growth in *A. melanogenum*. The high cell growth of *A. melanogenum* led to a high level of PMLA production. Therefore, we speculated that various nutritional substances provided by CSL benefit cell growth, which in turn benefit PMLA production.

With the help of metabolomics technology, the working mechanism underlying the effect of the addition of 3 g/L CSL on the metabolism of *A. melanogenum* was analyzed. The metabolomics data showed that the relative concentrations of metabolites involved in amino acid and organic acid metabolism were changed during fermentation (Fig. 5). The PLS-DA showed a clear separation between the two groups. Moreover, the tyrosine metabolism, glycine and serine metabolism, and TCA cycle were enhanced after the 3 g/L CSL addition. Among them, for the tyrosine metabolism, tyrosine first converted to 4-hydroxyphenylpyruvate then to homogentisate, which further converted to fumarylacetoacetate, and fumarylacetoacetate would transfer to fumarate that entered the TCA cycle (Wang et al. 2019). The metabolomics data showed that the relative concentration of homogentisate increased by 1.44-fold, and fumarate increased by 1.52-fold at 144 h compared to the control. The concentration of 5-aminolevulinate, related to the glycine and serine metabolism, increased about 1.2-fold. And 2-aminomuconate semialde hyde concentration increased about 1.3-fold, which can lead tryptophan into the glutaric acid pathway and then the TCA cycle (Fukuwatari et al. 2001). The result also proved that deoxyinosine, involved in purine metabolism, increased by 1.2-fold, and this metabolism is related to the cell’s nitrogen absorption that can provide molecules essential for DNA and RNA biosynthesis, energy metabolism, and signal transduction (Jessica and James 2017). The 5L-fermenter data showed that the maximal PMLA production was obtained after 120 h (Fig. 2a), but yield (Fig. 2e) was decreased after 120 h. These results were consistent with the metabolomics data that the metabolites reached the highest at 120 h then it began to decrease.

Metabolomics data can express the metabolic changes under different conditions. However, after the peak alignment and the data normalization, some of the target-metabolites may lose in the data. Therefore, we speculated that the concentrations of tyrosine, glycine, serine, and tryptophan, which related to the tyrosine pathway, glycine and serine pathway, and tryptophan pathway, were improved in the CSL group. These pathways may further increase the PMLA production. Consequently, we tested the nutritional substances of CSL and found 17 kinds of amino acids and three types of vitamins (Table 1). Among them, serine is 1.28%, tyrosine is 0.62%, tryptophan is 0.17%, and glycine is 2.05% of the total CSL weight.

In order to figure out which amino acid most benefiting PMLA production in the CSL, different amino acids related to the up-regulated pathway (Fig. 5) were added to the fermentation broth based on the ratio of their quality to the total CSL quality to evaluate the effect on PMLA production. The result demonstrated that these four amino acids increased PMLA production by 29.5% (tyrosine), 21.9% (serine), 9.3% (tryptophan), and 7.6% (glycine), respectively. Apart from that, a significant change between the tyrosine group and the control (P < 0.01) was observed, and this result is consistent with the enrichment analysis (Additional file 1: Figure S1), indicating that the CSL significantly influenced the tyrosine metabolism (P < 0.05). The data obtained from the PMLA-related pathway (Fig. 5) suggested that tyrosine flows to the TCA cycle by converted to fumarate, which is further converted to malic acid that increases the production of PMLA. Meanwhile, it also indicated that all of the four amino acids, tyrosine, glycine, serine, and tryptophan, eventually flows to the TCA cycle (Fig. 5). However, considering the relative concentration changes of the metabolites (pyruvate and fumarate), we can speculate that tyrosine may play a crucial role in increasing PMLA production under the CSL.

As mentioned above, the results showed that the TCA cycle was up-regulated after the addition of 3 g/L CSL, which would further increase PMLA production. Therefore, we can conclude that the up-regulated TCA cycle is the key metabolic pathway under the 3 g/L CSL for the increase of PMLA production among three speculated metabolic pathways, i.e., the glyoxylate acid pathway, the reductive TCA pathway, and the TCA pathway (Zou et al. 2019). Meanwhile, the energy provided by the up-regulated purine metabolism may accelerate this process.

The improvement in amino acid and organic acid metabolism in *A. melanogenum* from the addition of 3 g/L CSL generated various amino acids and organic acids to improve cell growth. The conversions of the metabolites related to the TCA cycle were enhanced after the addition of 3 g/L CSL. Therefore, we speculated that CSL is an effective stimulatory agent for cell growth and PMLA biosynthesis in *A. melanogenum*. Meanwhile, CSL could be used as an economic nitrogen source due to its high nutrition and low cost. As a potential replacement of peptone and yeast extract in PMLA production, CSL has satisfactory use prospects in the PMLA industry.

## Supplementary information

**Additional file 1:** **Figure S1.** Enrichment analysis of metabolomics data.

## Data Availability

All data generated or analyzed during this study are included in this paper.
