# Supplementary material for: Effects of corn steep liquor on β-poly(l-malic acid) production in Aureobasidium melanogenum
Source: AMB Express. 2020 Dec 1;10:211. doi: 10.1186/s13568-020-01147-8 (PMC7708538; doi:10.1186/s13568-020-01147-8)
Supplement: Supplementary file 1 — Additional file 1: Figure S1. Enrichment analysis of metabolomics data. [file 13568_2020_1147_MOESM1_ESM.pdf]

Additional file 1

AMB Express

Effects of corn steep liquor on ß-poly(L-malic acid) production in *Aureobasidium melanogenum*

Genan Wang [1,2], Bingyi Shi [1,2], Pan Zhang [1,2], Tingbin Zhao [4], Haisong Yin [1,2,3,*], Changsheng Qiao [1,2,4,*]

1.Key Laboratory of Industrial Fermentation Microbiology (Tianjin University of Science and Technology), Ministry of Education, Tianjin, 300457, P.R.China

2.Tianjin Engineering Research Center of Microbial Metabolism and Fermentation Process Control, College of Biotechnology, Tianjin University of Science and Technology, Tianjin 300457, P.R.China

3.School of Bioengineering, Tianjin Modern Vocational Technology College, Tianjin 300350, P.R.China

4. Tianjin Huizhi Biotrans Bioengineering Co., Ltd. Tianjin 300457, P.R.China

* Corresponding author.

Changsheng Qiao, Tel: +86 139-2004-8605, E-mail: qiaochangsheng@163.com.

Haisong Yin: Tel: +86 175-2695-2844, E-mail: 371954887@qq.com

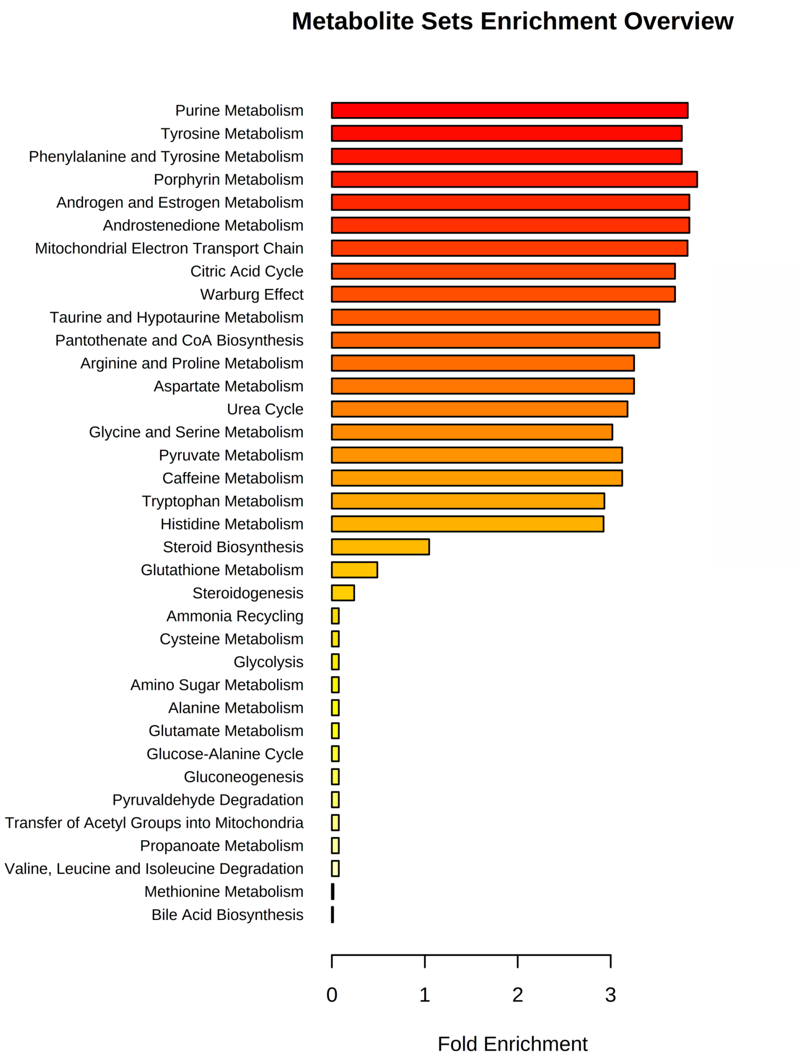

Figure S1 Enrichment analysis of metabolomics data
